# Colonization–competition dynamics of basal species shape food web complexity in island metacommunities

**DOI:** 10.1007/s42995-023-00167-0

**Published:** 2023-05-09

**Authors:** Guanming Guo, Fei Zhao, Ivan Nijs, Jinbao Liao

**Affiliations:** 1 Ministry of Education’s Key Laboratory of Poyang Lake Wetland and Watershed Research, School of Geography and Environment, Jiangxi Normal University, Nanchang, 330022 China; 2 Research Group in Plants and Ecosystems, Department of Biology, University of Antwerp, 2610 Wilrijk, Belgium

**Keywords:** Competition–colonization tradeoff, Food web complexity, Hierarchical competition, Patch-dynamic framework, Patch loss

## Abstract

Exploring how food web complexity emerges and evolves in island ecosystems remains a major challenge in ecology. Food webs assembled from multiple islands are commonly recognized as highly complex trophic networks that are dynamic in both space and time. In the context of global climate change, it remains unclear whether food web complexity will decrease in a monotonic fashion when undergoing habitat destruction (e.g., the inundation of islands due to sea-level rise). Here, we develop a simple yet comprehensive patch-dynamic framework for complex food web metacommunities subject to the competition-colonization tradeoff between basal species. We found that oscillations in food web topological complexity (characterized by species diversity, mean food chain length and the degree of omnivory) emerge along the habitat destruction gradient. This outcome is robust to changing parameters or relaxing the assumption of a strict competitive hierarchy. Having oscillations in food web complexity indicates that small habitat changes could have disproportionate negative effects on species diversity, thus the success of conservation actions should be evaluated not only on changes in biodiversity, but also on system robustness to habitat alteration. Overall, this study provides a parsimonious mechanistic explanation for the emergence of food web complexity in island ecosystems, further enriching our understanding of metacommunity assembly.

**Supplementary Information:**

The online version contains supplementary material available at 10.1007/s42995-023-00167-0.

## Introduction

How complexity arises and persists in natural communities has been a central issue in ecology (Allesina and Tang 2012; May 1972; McCann 2000). Early theoretical studies have shown that complex food webs are unlikely to persist, as complexity tends to destabilize population dynamics (Chen and Cohen 2001; Gilpin 1975; May 1972). However, the apparent contradiction between theory and observation (Pimm 1991) has stimulated theoretical work seeking a mechanism for the maintenance of complex food webs (DeAngelis 1975; Kondoh 2003; McCann et al. 1998; Neutel et al. 2002; Yodzis 1981). There are many different approaches to model food web complexity, each emphasizing different factors by which food web structure might be controlled. For example, many models have highlighted the importance of realistic network topologies (Martinez et al. 2006; Yodzis 1981), non-random patterns of interaction strengths (Berlow et al. 2004; Gross et al. 2009; McCann et al. 1998; Neutel et al. 2002), effects mediated by natural body size (Brose et al. 2006; Kartascheff et al. 2010; Yodzis and Innes 1992), and foraging adaptation (Beckerman et al. 2006; Cattin et al. 2004; Kondoh 2003; Petchey et al. 2008). These studies have greatly advanced our understanding of the food web complexity–stability relationship. However, these non-spatial models have primarily focused on local-scale trophic dynamics, ignoring the role of space in structuring food webs on the landscape scale.

In nature, many communities consist of relatively isolated subcommunities, linked by species dispersal, within a landscape (Fortuna and Bascompte 2006; Gravel et al. 2011, 2016; Jabot and Bascompte 2012; Liao et al. 2017a, b, c, 2020a; Pillai et al. 2010, 2011). As such, numerous models have explored the relationship between interaction network structure and metacommunity persistence using the patch-dynamic framework (Albouy et al. 2019; Baiser et al. 2019; Galiana et al. 2018; Grass et al. 2018; Guimarães Jr. 2020; Liao et al. 2020b, 2022a; McWilliams et al. 2019; Poisot et al. 2014; Schleuning et al. 2016; Staniczenko et al. 2017). This theoretical framework allows for a spatial perspective on ecological networks by viewing them as the regional assembly of simpler, spatially distributed subnetworks (Pillai et al. 2010). Interestingly, Pillai et al. (2011) used the patch-dynamic framework to show that as habitat destruction increases, food web complexity and species diversity may increase by the structural role played by network branches that are supported by omnivore and generalist feeding links. Furthermore, Liao et al. (2017c) demonstrated that even in a simple trophic module with a dispersal-competition tradeoff between two species, intermediate levels of habitat destruction can enhance biodiversity and, therefore, trophic complexity by creating refuges for the poorer competitor. More recently, Liao et al. (2022b) found that in non-trophic communities, the interaction of habitat disturbance and competition–colonization (C–C) tradeoffs can yield strong oscillations in biodiversity along the disturbance gradient. However, whether and how these complex responses can cascade through the entire complex food web remain unclear. In this study, we develop a simple but comprehensive patch-dynamic framework for complex trophic systems subject to the C–C tradeoff between basal species. Using this framework, we check whether food web complexity will oscillate when undergoing habitat destruction (e.g., the inundation of islands), a key driver of biodiversity loss (Pimm and Raven 2000).

## Results

**Fig. 1:**
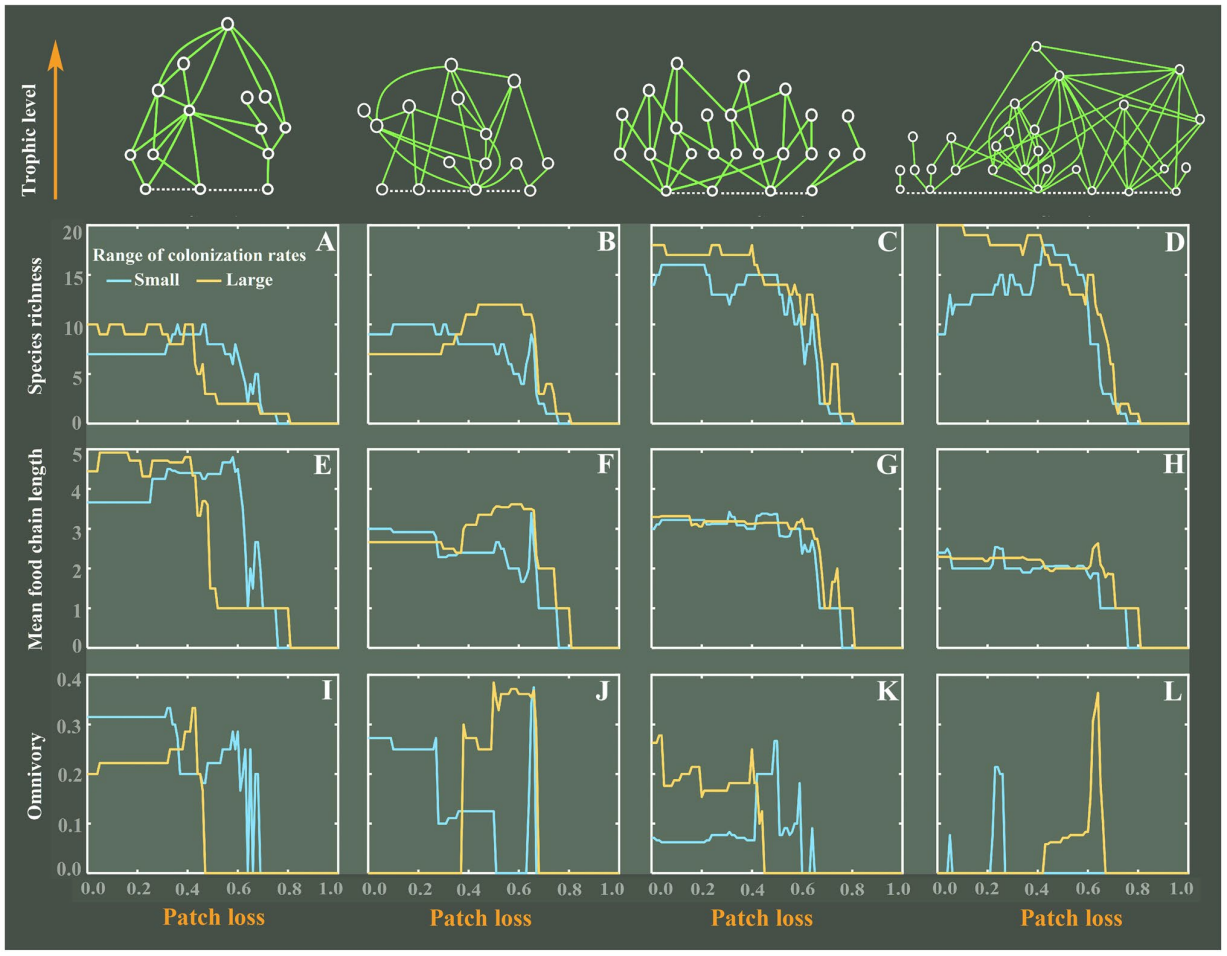
Effect of patch loss (*U* – the proportion of permanently destroyed patches) on the complexity of empirical food webs (cases 1 ~ 4) at steady state, characterized by species richness, mean food chain length and omnivory. A strict hierarchical competition among basal species is considered by ranking them from the best competitor (species 1) to the poorest (species $${n}_{P}$$), i.e., *H*_*ij*_ = 1 for *i* < *j* and 0 otherwise, in a competitive matrix ***H***. To set up the competition–colonization tradeoff, basal species colonization rates are evenly spaced in increasing order at both small ($${c}_{i}^{P}$$ ϵ*E*[0.45, 0.8]) and large ($${c}_{i}^{P}$$ ϵ*E*[0.25, 1]) ranges, while their extinction rates are fixed at $${e}_{i}^{P}$$= 0.2. Other parameters: colonization rates of consumers $${c}_{i}^{A} $$= 0.625, extinction rates $${e}_{i}^{A} $$ = 0.05, top-down extinction rates of both basal and consumer species (due to over-predation) are equal with $${\mu }_{ik}={\varphi }_{ik} $$= 0.05

We first implemented a basic numerical simulation for the initial four empirical food webs from island ecosystems (Fig. 1) by considering the C–C tradeoff among basal species in a strict competitive hierarchy. More specifically, basal species were ranked from the best competitor to the poorest, while colonization rate was negatively correlated with competition ability. These four empirical food webs, observed from diverse island ecosystems, show different structural properties (Table 1), characterized by mean food chain length (FCL), max FCL, species diversity (*S* = ${n}_{P}+{n}_{A}$, with ${n}_{P}$—the number of basal species and ${n}_{A}$—the number of consumers), basal species richness (${n}_{P}$), the total number of trophic links (*L*), connectance ($C=2L/\left[S\times\left(S-1\right)\right]$), and the degree of omnivory. Although the overall trend in food web complexity (including species richness, mean FCL and omnivory) is monotonically decreasing, a mixture of weak and strong oscillations in these structural properties emerge along the gradient of patch loss, under either scenario where the range of basal species’ colonization rates is small or large (Fig. 1). In particular, such oscillations in the degree of omnivory are stronger than the other two structural metrics. Finally, we observed that having no patch loss does not guarantee the highest species richness, the largest mean FCL and the highest degree of omnivory. This outcome demonstrates that food web complexity does not decrease in a simple monotonic fashion along the habitat destruction gradient, instead increasing habitat destruction may shape more complex food webs.

**Table 1:** Structural properties of four empirical food webs from island ecosystems (shown in Fig. 1; FCL—food chain length)

Food webs Properties	Case 1	Case 2	Case 3	Case 4
Mean FCL	3.56	2.44	2.30	3.69
Max FCL	6	5	4	8
No. species (*S*)	14	15	24	28
No. basal species	3	4	4	6
No. links (*L*)	23	25	34	55
Connectance *C* = 2*L*/[*S*×(*S*‒1)]	0.25	0.24	0.12	0.15
Omnivory	0.29	0.40	0.21	0.36
Location	Coral reefs Marshall Islands	Salt marsh Rhode Island	Salt marsh Long Island	Spitsbergen Bear Island
Reference	Hiatt and Strasburg (1960)	Nixon and Oviatt (1973)	Woodwell (1967)	Summerhayes and Elton (1923)

**Fig. 2:**
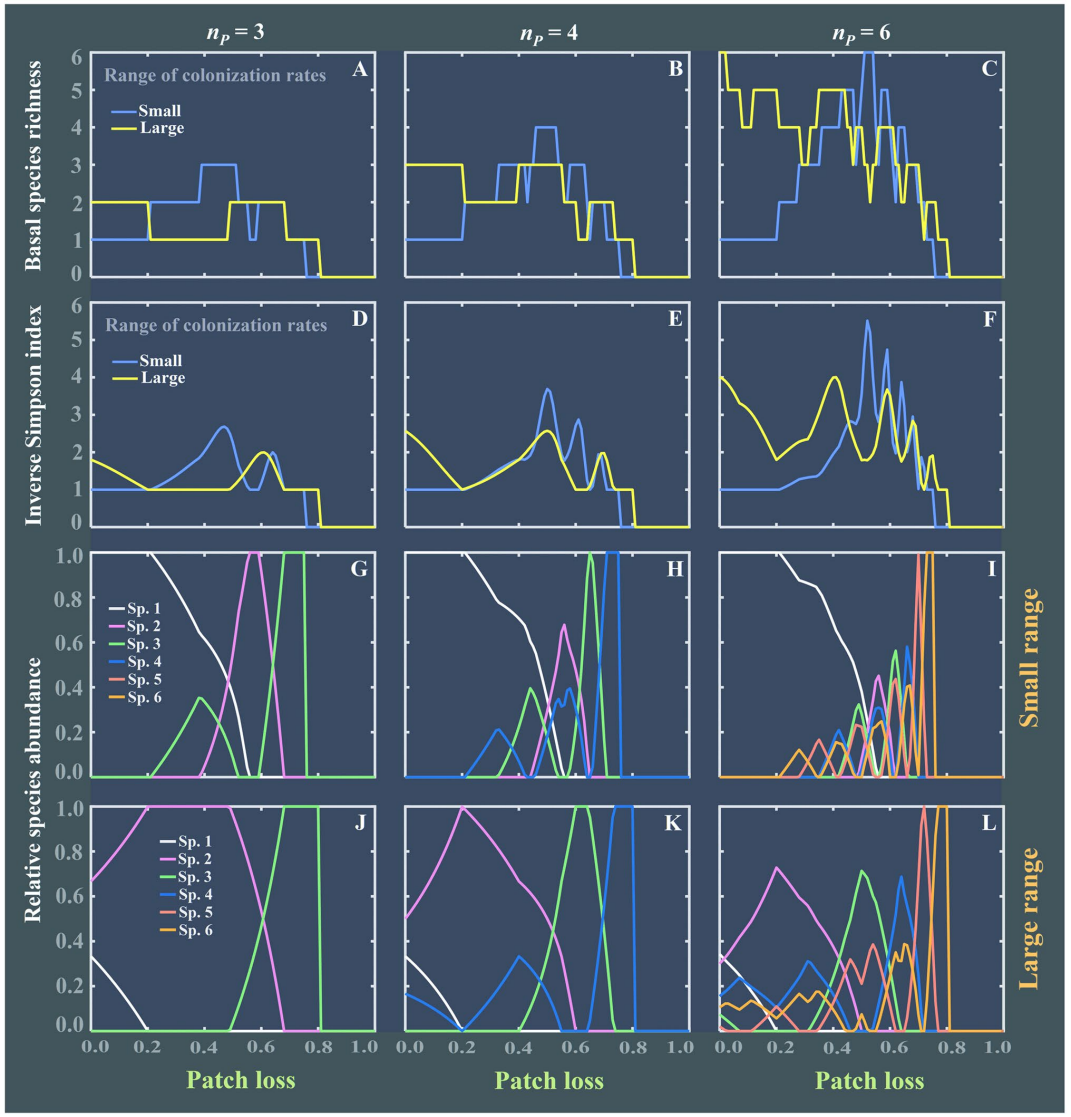
Effect of patch loss (*U*) on basal species diversity (**A**–**F**) and their relative abundances (**G**–**L**) at steady state, while ignoring the top-down effect from consumers (i.e., ${\mu }_{ik}=0$). Basal species diversity is characterized by both basal species richness and the inverse Simpson index ($1/\sum {q}_{i}^{2}$, with ${q}_{i}={P}_{i}/\sum {P}_{j}$ being the relative abundance of basal species *i*). Initial basal species richness is set as ${n}_{P}=$ 3, 4 or 6. Other parameter settings are the same as in Fig. 1

To illustrate the complex response of these empirical food webs to patch loss, we further considered how the diversity and relative abundances of basal species vary with increasing patch loss, while ignoring the top-down effect (i.e., no predation term in Eq. 1). As shown in Fig. 2, basal species diversity, characterized by species richness and the inverse Simpson index ($1/\sum {q}_{i}^{2}$, with ${q}_{i}={P}_{i}/\sum {P}_{j}$ being species relative abundance at steady state) rises and falls several times along the gradient of patch loss at both small and large ranges of colonization rates. This means that increasing patch loss can yield multiple peaks in basal species diversity, with more peaks emerging in species-richer communities (Fig. 2A-F). Like in Fig. 1, having no patch loss does not yield the most diverse communities of basal species, while intermediate levels of patch loss might result in the highest basal species diversity. The level of patch loss at which a basal species enters or leaves the system is a “turning point” (Fig. 2G-L), i.e., those low abundant species that have been declining start to increase, while species with high abundance begin to decrease, therefore forming a zig–zag pattern. Whenever some basal species are high in relative abundance but others are low, basal species diversity is low due to extreme unevenness. In contrast, whenever basal species’ relative abundances are similar, basal species diversity is boosted by high evenness. Thus, such a pattern would naturally lead to an oscillating diversity profile. Due to the bottom-up control, basal species abundances can determine the persistence of their associated consumers at higher trophic levels, i.e., the survival of consumers depends on the associated basal species (see Eq. 6 in *Materials and Methods*). Therefore, such oscillating patterns in basal species diversity would propagate to higher trophic levels and change the food web structure, thereby shaping oscillations in the food web complexity shown in Fig. 1.

These oscillating patterns in food webs were also robust to relaxing model assumptions, such as evenly spaced colonization rates and a strict competitive hierarchy among basal species (Supplementary Figs. S1-S4). For instance, as patch loss increases, oscillations in species richness, mean FCL and omnivory also occur in food webs with irregularly spaced colonization rates of basal species (Supplementary Fig. S1). Furthermore, when weakening a strict competitive hierarchy (Supplementary Figs. S1–S2), we also observed oscillations in food web complexity though they eventually decreased. Even when the competitive hierarchy was violated (i.e., when basal species’ competition abilities were structured as perfectly intransitive competition), these oscillating patterns were still observed (compare hierarchical competition *RI* = 0 with intransitive competition *RI* = 1 in Supplementary Fig. S3). Nevertheless, intransitive competition generally yields somewhat less pronounced oscillating patterns than hierarchical competition. This is probably due to the fact that we did not impose a global C–C tradeoff on basal species in these simulations; instead, local C–C tradeoffs, involving only a subset of the basal species, were created at random (compare Supplementary Fig. S4G-I with Fig. 2J-L). As such, the overall oscillating pattern in basal species diversity is weaker than that obtained for strict hierarchical competition, where the C–C tradeoff applies to all basal species (compare Supplementary Fig. S4A-F with Fig. 2A-F; yellow curves). Thus, intransitive competition with local C–C tradeoffs generally results in weaker oscillations in food web complexity than hierarchical competition with global C–C tradeoffs.

## Discussion

In this study, we show that oscillations in food web complexity emerge along the habitat destruction gradient. This outcome is robust to a broad range of model assumptions and parameter choices, with the only necessary assumption being a C–C tradeoff between basal species. This further confirms the importance of habitat destruction in controlling food web structure (McHugh et al. 2010; Post 2002). Essentially, the emergence of oscillations in food web complexity originates from the interaction of habitat destruction and C–C tradeoffs among basal species. This interaction can facilitate different subsets of basal species to coexist and, therefore, alter food web structure via bottom-up control. More specifically, a basal species leaving the trophic system (i.e., going extinct) would trigger secondary extinctions for its directly or indirectly associated consumers, thereby reducing vertical species diversity and simplifying food web structure. In contrast, a basal species entering the trophic system would support the survival of its associated consumers, which can promote vertical biodiversity and, therefore, complicate food web topology. Thus, as habitat destruction increases, the alternating pattern of basal species entering or leaving the trophic system (similar to Tilman et al. 1997) ultimately results in oscillations in overall food web complexity due to trophic cascading effects.

It should be emphasized that the trophic and non-trophic interactions among species in a given metacommunity can be a key determinant of the effects of habitat destruction (Amarasekare 2008; Gonzalez et al. 2011; Holt 2002; Pillai et al. 2011). According to the trophic rank hypothesis (Kruess and Tscharntke 1994), species at higher trophic levels are the first to go extinct as habitat loss increases. However, omnivores do not necessarily follow this paradigm, because they can switch feeding on different trophic-level prey species for survival (Liao et al. 2017a, b, 2020a; Melián and Bascompte 2002; Pillai et al. 2011). Furthermore, the indirect interaction between species, such as exploitative or apparent competition, can also modify the sensitivity of their predators to habitat destruction (Liao et al. 2017b; Melián and Bascompte 2002). In particular, the emergence of oscillating patterns in our model requires the potential food web to be sufficiently complex, otherwise such oscillations would not be observed (e.g., in networks consisting primarily of one or more parallel food chains). Therefore, food web complexity is not only determined by C–C dynamics and landscape properties, but also by the trophic structure in which the species are embedded.

The complex response of food webs to habitat destruction suggests a change in perspective. Our argument is that the noisiness of the responses of food web complexity to habitat destruction in empirical studies might not simply reflect sampling artifacts, experimental error, transient effects, or stochasticity, as thought previously. Instead, it arises deterministically from the C–C dynamics in complex trophic systems. This raises the possibility that typical activities of biodiversity conservation (e.g., habitat restoration and/or increasing habitat connectivity) bring the risk of further species losses if carried out without first analyzing their potential consequences for the whole trophic system. Thus, we advise caution when designing conservation strategies for community biodiversity. Identification of the trophic structures, species demographic traits, competition abilities and landscape properties from empirical data are essential precursors to setting conservation priorities in applied ecology. Furthermore, biodiversity, the goal of conservation, is not necessarily itself a good measure of conservation success. Given the oscillatory relationship between biodiversity and habitat destruction in a complex trophic system, an observed burst in biodiversity does not mean that the system would be able to tolerate even more habitat destruction. In fact, according to our model, a system that is near catastrophic collapse may experience sudden biodiversity growth in response to habitat destruction before any further habitat loss induces the actual demise. Thus, biodiversity and system robustness to habitat alteration, rather than biodiversity alone, are required to evaluate conservation success. At present, many marine ecosystems worldwide that are structured primarily by basal species (e.g., seagrass meadows, salt marshes and coral reefs) are declining at an alarming rate due to anthropogenic disturbances (Bellard et al. 2012; Borst et al. 2018; Gedan et al. 2009; Waycott et al. 2009). Our finding of the importance of C–C dynamics among basal species in controlling food web structure suggests that to preserve complex but stable food webs across ecosystems, it is also vital to prioritize the conservation and restoration of the basal species that support them.

In conclusion, we provide a simple yet robust conceptual metacommunity framework to show that habitat destruction can lead to oscillations in food web complexity. This study demonstrates the importance of the interaction between habitat destruction and C–C dynamics in shaping food web complexity. Thus, extending the patch-dynamic metacommunity framework to complex food webs is a critical step in the development of a unified theory of biodiversity. Such a unified theory would provide an explicit principle of how food webs assemble and disassemble in space and how their complexity varies with habitat destruction at a spatial scale. Experimental tests of our results are possible with natural or laboratory-based model systems that allow the direct manipulation of metacommunity size and connectivity (Gonzalez et al. 1998). Overall, this study offers a parsimonious explanation for the emergence of food web complexity in fragmented landscapes, further enriching our understanding of metacommunity responses to habitat destruction.

## Materials and methods

### Modeling framework

We consider an ecosystem consisting of a large number of patches (i.e., islands) where each patch can accommodate one subpopulation of each species and all species within the food web can disperse randomly across all patches. Yet, we particularly assume that competition among basal species (at the first trophic level) can occur immediately through displacement of an inferior resident by a superior competitor (*competitive displacement*; cf. Tilman 1994; Tilman et al. 1994, 1997), thus different basal species cannot coexist stably in the same patch. Based on previous work (Hastings 1980; Li et al. 2020; Liao et al. 2022b; Tilman 1994; Tilman et al. 1994, 1997), we can write the patch occupancy dynamics of basal species subject to the *colonization–extinction–competition–predation* processes

1 $$ \frac{{dP_{i} }}{dt} = \underbrace {{c_{i}^{P} P_{i} \left( {1 - U -  {\sum} _{j = 1}^{{n_{P} }} P_{j} } \right)}}_{Colonization}\underbrace {{ - e_{i}^{P} P_{i} }}_{Extinction}\underbrace {{ { + \sum }_{j = 1}^{{n_{P} }} \left( {c_{i}^{P} P_{i} H_{ij} P_{j} - c_{j}^{P} P_{j} H_{ji} P_{i} } \right)}}_{Competitive\, displacement}\underbrace {{ - P_{i}  {\sum} _{k = 1}^{{n_{A} }} \theta_{ik} \mu_{ik} A_{k} }}_{{Top - down}\; {predation}}, $$

where ${P}_{i}$ and ${A}_{k}$ separately represent the patch occupancies of basal species *i* and consumer *k*, ${c}_{i}^{P}$ and ${e}_{i}^{P}$ are the colonization and intrinsic extinction rates of basal species *i* separately, and ${n}_{P}$ (or ${n}_{A}$) is the number of basal species (or consumers) in the food web. The competition strength of basal species *i* compared to basal species *j* is ${H}_{ij}$, which is encoded in a competitive matrix ***H***. The top-down extinction rate of basal species *i* due to over-predation by consumer *k* is ${\mu }_{ik}$, while ${\varvec{\theta}}$ is the interaction matrix, with ${\theta }_{ik}=1$ if consumer *k* can feed on basal species *i* (otherwise ${\theta }_{ik}$=0).

The *colonization* term describes the rate at which species *i* can colonize those patches unoccupied by any basal species $\left(1-U-\sum_{j=1}^{{n}_{P}}{P}_{j}\right)$, with $U$ being patch loss (defined as the proportion of permanently destroyed patches that are unsuitable for species colonization, such as the loss of islands due to sea-level rise). The total number of colonizers (e.g., propagules) produced by basal species *i* is proportional to its overall population size (${c}_{i}^{P}{P}_{i}$). The *extinction* term is relatively straightforward: subpopulations of basal species *i* are assumed to become extinct with a rate ${e}_{i}^{P}$, thus the overall population loss for basal species *i* should be ${e}_{i}^{P}{P}_{i}$. Similarly, the *predation* term is the sum of increased extinction of basal species *i* due to over-predation by different consumers, with the encounter rate linearly related to ${{P}_{i}A}_{k}$.

The *competition* term describes competitive displacement between basal species, that is, colonizers from one species (${c}_{i}^{P}{P}_{i}$ or ${c}_{j}^{P}{P}_{j}$) arrive at a patch already occupied by another species and displace it. The displacement probability depends on the relative competition strength (${H}_{ij}$) of the species involved. In particular, the parameters ${H}_{ij}$ and ${H}_{ji}$ are the independent probabilities that a subpopulation of species *i* displaces species *j* and that a subpopulation of species *j* displaces species *i*, respectively. In fact, both parameters ${H}_{ij}$ and ${H}_{ji}$ can characterize much more complex competition structures, for example, a strict hierarchical competition (Tilman1994; Tilman et al. 1994, 1997) by setting ${H}_{ij}=1$ if *i* < *j* and 0 otherwise (Liao et al. 2022b), or intransitive competition by perturbing the hierarchical competition matrix ***H*** (Li et al. 2020; Rojas-Echenique and Allesina 2011). The net change in the population size of species *i*, because of displacement competition with species* j* is given by ${P}_{i}{P}_{j}({c}_{i}^{P}{H}_{ij}-{c}_{j}^{P}{H}_{ji})$. Therefore, the competition term is the sum of the net result of pairwise competition events, depending on the colonization pressure exerted by these basal species (cf. Li et al. 2020; Liao et al. 2022b).

Then, we characterize the patch dynamics of consumers (including top predators) in the food web. For model simplicity, we assume that different consumers can co-occur in the same patch by ignoring their competition, and a consumer species has the same colonization rate when feeding on different prey species. Thus, we can write the patch occupancy dynamics for consumer *i* subject to the *colonization–extinction–predation* processes

2 $$ \frac{{dA_{i} }}{dt} = \underbrace {{c_{i}^{A} A_{i} \left( {{ \sum} _{j = 1}^{{n_{P} }} \theta_{ji} P_{j} +  {\sum} _{k = 1}^{{n_{A} }} \delta_{ki} A_{k} } \right)\left( {1 - U - A_{i} } \right)}}_{Colonization}\underbrace {{ - e_{i}^{A} A_{i} }}_{Extinction}\underbrace {{ - A_{i} {\sum} _{k = 1}^{{n_{A} }} \varphi_{ik} \delta_{ik} A_{k} }}_{Predation}, $$

where ${c}_{i}^{A}$, ${e}_{i}^{A}$ and ${\varphi }_{ik}$ are the colonization rate, intrinsic extinction rate and top-down extinction rate (due to over-predation by another consumer *k*) of consumer *i*, respectively. Similar to basal species, in the *colonization* term, $\left(1-U-{A}_{i}\right)$ is the fraction of suitable patches that are unoccupied by consumer *i*, and ${{c}_{i}^{A}A}_{i}\left(\sum_{j=1}^{{n}_{P}}{{\theta }_{ji}P}_{j}+\sum_{k=1}^{{n}_{A}}{{\delta }_{ki}A}_{k}\right)$ is the total number of colonizers produced by consumer *i* when feeding on different prey species (including both basal and consumer species), with ${A}_{i}{P}_{j}$ or ${A}_{i}{A}_{k}$ being the encounter rate. If consumer *i* can feed on basal species *j* (or another consumer *k*), then ${\theta }_{ji}=1$ (or ${\delta }_{ki}=1$), and 0 otherwise. The *predation* term describes the total population loss of consumer *i* being eaten by different predators. Top predators do not suffer from top-down predation, thus their patch dynamics lack the *predation* term present in Eq. (2).

### Model properties

For mathematical tractability, we disregard the top-down predation in Eqs. (1 and 2). As such, Eq. (1) can be rearranged as

3 $$ \frac{{dP_{i} }}{dt} = P\left[ {\underbrace {{c_{i}^{P} \left( {1 - U} \right) - e_{i}^{P} }}_{{b_{i} }} + \underbrace {{{\sum} _{j = 1}^{{n_{P} }} \left( {c_{i}^{P} H_{ij} - c_{j}^{P} H_{ji} - c_{i}^{P} } \right)P_{j} }}_{{M_{ij} }}} \right]. $$

In this formulation, ${b}_{i}$ is the effective intrinsic growth rate of basal species *i*, while ${M}_{ij}$ is the effective interaction coefficient (including intra- and inter-specific competition). The net effect of these two terms in the square bracket is the *per-capita* growth rate ${r}_{i}=\frac{1}{{P}_{i}}\frac{d{P}_{i}}{dt}$ of basal species *i*. In particular, the *per-capita* growth rate is linear in ${P}_{i}$ and has the Lotka-Volterra form ${r}_{i}={b}_{i}+{\sum }_{j=1}^{{n}_{P}}{M}_{ij}{p}_{j}$. Therefore, it has at most one fixed point where all species’ patch occupancies ${P}_{i}^{*}$ are positive (i.e., a coexistence steady state). This steady state can be written as

4 $${P}_{i}^{*}=-{\sum }_{j=1}^{{n}_{P}}{{b}_{j}\left({{\varvec{M}}}^{-1}\right)}_{ij}=-{\sum }_{j=1}^{{n}_{P}}{\left({{\varvec{M}}}^{-1}\right)}_{ij}\left[{c}_{j}^{P}\left(1-U\right)-{e}_{j}^{P}\right],$$

where ${\left({{\varvec{M}}}^{-1}\right)}_{ij}$ is the $\left(i,j\right)$ th entry of the inverse of the effective interaction matrix ${\varvec{M}}$. Moreover, if the tournament matrix ***H*** is fully hierarchical (${H}_{ij}=1$ if $i<j$ and 0 otherwise), the feasible equilibrium point in which the most species survive is stable (similar to stability analysis for competitive communities in Hastings 1980; Liao et al. 2022b; Tilman et al. 1997).

By ignoring the predation term in Eq. (2), we have

5 $$\frac{d{A}_{i}}{dt}={A}_{i}\left[{c}_{i}^{A}\left({\sum} _{j=1}^{{n}_{P}}{{\theta }_{ji}P}_{j}+{\sum} _{k=1}^{{n}_{A}}{{\delta }_{ki}A}_{k}\right)\left(1-U-{A}_{i}\right)-{e}_{i}^{A}\right].$$

Given that the equilibrium point is feasible, we can express the patch occupancies for all consumers at equilibrium as

6 $${A}_{i}^{*}=1-U-\frac{{e}_{i}^{A}}{{c}_{i}^{A}\left(\sum_{j=1}^{{n}_{P}}{\theta }_{ji}{P}_{j}^{*}+\sum_{k=1}^{{n}_{A}}{\delta }_{ki}{A}_{k}^{*}\right)},$$

in which ${P}_{j}^{*}$ is already determined from Eq. (4), independent of the patch dynamics of consumers. If ${\delta }_{ki}=1$ (i.e., consumer *i* can feed on consumer *k*), then ${A}_{i}^{*}$ is related to ${A}_{k}^{*}$, but ${A}_{k}^{*}$ is irrelevant to ${A}_{i}^{*}$ (as these food webs exclude loops and cannibalism). When ${\delta }_{ki}=0$, the equilibrium patch occupancies of both consumers *i* and *k* are mutually independent as we assume that there are no top-down effects in the whole trophic system. Therefore, the survival of consumer *i* depends on its prey species abundances.

### Numerical analysis

We use the theoretical framework outlined above to analyze how patch loss affects food web structure. From the large number of indices that can characterize food web complexity, we select three that are typical: species richness (i.e., the total number of species in the food web), mean food chain length (mean FCL; i.e., the mean of all food chain lengths in the food web, with a food chain being a linked path from a top predator to a basal species), and omnivory (i.e., the fraction of species that consume two or more species and have food chains of different lengths). Basal species are ranked according to their colonization rates, so that species 1 has the lowest colonization rate and species ${n}_{P}$ has the highest (i.e., ${c}_{1}^{P}<{c}_{2}^{P}<\dots <{c}_{{n}_{P}}^{P}$). Initially, we assume a strict competitive hierarchy by ranking the basal species from the best competitor (species 1) to the poorest (species ${n}_{P}$). This might set up a classic competition–colonization (C–C) tradeoff among basal species, where colonization rate is negatively correlated with competition ability (Tilman 1994). Next, we also consider basal species with perfect intransitive competition, i.e., relative intransitivity *RI* = 1 (e.g., a rock-paper-scissors game for a system with three competitors) by perturbing the strict hierarchical competition matrix ***H*** (see details in Rojas-Echenique and Allesina 2011), while retaining the ranking of basal species by colonization rate.

We first take four empirical food webs observed from island ecosystems as the initial webs in our model (see details in Table 1; illustrated in Fig. 1). Subsequently, we apply numerical methods (with ODE45 in Matlab R2016a) to determine the species abundances (patch occupancies) at steady state. To reach the steady state, initially we run each case for a long time and find that initial species abundances do not affect system steady state. Based on numerous preliminary trials, 15,000 time units are sufficient for all cases to achieve steady state. We simulate the patch dynamics for a further 5,000 time units and take the average patch occupancy of each species across this period to be an estimate of the steady-state species abundances and therefore food web structure (note that a species is deemed extinct if its abundance drops below 10^–6^).

## Supplementary Information

Below is the link to the electronic supplementary material.

Supplementary file1 (DOCX 925 KB)

## Data Availability

This theoretical study produced no new data, but the novel code used to model the effect of patch loss on food web complexity is available on Zenodo at https://doi.org/10.5281/zenodo.7244269.
