# Supplementary material for: Colonization–competition dynamics of basal species shape food web complexity in island metacommunities
Source: Mar Life Sci Technol. 2023 May 9;5(2):169–77. doi: 10.1007/s42995-023-00167-0 (PMC10232389; doi:10.1007/s42995-023-00167-0)
Supplement: Supplementary file 1 — Supplementary file1 (DOCX 925 KB) [file 42995_2023_167_MOESM1_ESM.docx]

**Supplementary Information**

**Colonization-competition dynamics of basal species shape food web complexity in island metacommunities**

Guanming Guo^1^, Fei Zhao^1^, Ivan Nijs^2^, Jinbao Liao^1,*^

^1^Ministry of Education’s Key Laboratory of Poyang Lake Wetland and Watershed Research, School of Geography and Environment, Jiangxi Normal University, Nanchang 330022, China

^2^Research group Plants and Ecosystems, Department of Biology, University of Antwerp, Wilrijk 2610, Belgium

*Corresponding author: [jinbaoliao@163.com](mailto:jinbaoliao@163.com) (J.L.)

**

**

**Supplementary Figure S1.** Effect of patch loss on the complexity of four empirical food webs (as shown in Fig. 1) at steady state, characterized by species richness (**A-D**), mean food chain length (**E-H**) and omnivory (**I-L**). Two cases are considered: First, basal species colonization rates are evenly spaced over a large range ($c_{i}^{P}$ϵ*E*[0.25, 1]) while weakening their competitive hierarchy ***H***: the upper and lower triangular entries (*H_ij_*) are uniformly sampled from *U*[0.75, 1] and *U*[0, 0.25] separately (blue lines). Second, basal species colonization rates are uniformly drawn from a large range $c_{i}^{P}$ϵ*U*[0.25, 1] and sorted in increasing order, but with a strict competitive hierarchy *H* ($H_{ij}=1$ for *i<j* and 0 otherwise; yellow lines). Other parameters are the same as in Fig. 1.

**

**

**Supplementary Figure S2.** Effect of patch loss on the complexity of four empirical food webs (shown in Fig. 1) at steady state, characterized by species richness (**A-D**), mean food chain length (**E-H**) and omnivory (**I-L**). Basal species’ colonization rates are evenly spaced over a large range ($c_{i}^{P}$ϵ*E*[0.25, 1]) while gradually weakening their competitive hierarchy ***H***: the upper triangular entries are all set as *H_ij_*=1, 0.8 or 0.6, corresponding to the lower triangular entries *H_ij_*=0, 0.2 or 0.4, respectively. Other parameters are the same as in Fig. 1.

**

**

**Supplementary Figure S3.** Effect of patch loss on four empirical food webs (as shown in Fig. 1) at steady state, by comparing two types of competition among basal species: a strict hierarchical competition (i.e., *H_ij_*=1 for *i<j* and 0 otherwise in a competitive matrix ***H***, with relative intransitivity *RI*=0), and perfect intransitive competition by perturbing the strict competitive hierarchy (*RI*=1 with the competitive matrix ***H*** displayed in response to four empirical food webs). Basal species colonization rates are evenly spaced over a large range ($c_{i}^{P}$ϵ*E*[0.25, 1]). Other parameters: see Fig. 1.

**

**

**Supplementary Figure S4.** Effect of patch loss on basal species diversity (**A-F**) and their relative abundances (**G-I**) at steady state under perfect intransitive competition (*RI*=1, as shown in the competitive matrix ***H***), while ignoring the top-down effect from consumers (i.e., $\mu_{ik}=0$). Basal species diversity is characterized by both basal species richness and the inverse Simpson index ($1/{\sum q_{i}^{2}}$, with $q_{i}=P_{i}/\sum P_{j}$ being the relative abundance of basal species *i*). Initial basal species richness is set as $n_{P}=$3, 4 and 6. Other parameters are the same as in Fig. S3 above.
